# Candidate SNP markers of changes in the expression levels of the human SCN9A gene as a hub gene for pain generation, perception, response and anesthesia

**DOI:** 10.18699/vjgb-24-89

**Published:** 2024-12

**Authors:** P.A. Dotsenko, K.A. Zolotareva, R.A. Ivanov, I.V. Chadaeva, N.L. Podkolodnyy, V.A. Ivanisenko, P.S. Demenkov, S.A. Lashin, M.P. Ponomarenko

**Affiliations:** Institute of Cytology and Genetics of the Siberian Branch of the Russian Academy of Sciences, Novosibirsk, Russia Novosibirsk State University, Novosibirsk, Russia Kurchatov Genomic Center of ICG SB RAS, Novosibirsk, Russia; Institute of Cytology and Genetics of the Siberian Branch of the Russian Academy of Sciences, Novosibirsk, Russia; Institute of Cytology and Genetics of the Siberian Branch of the Russian Academy of Sciences, Novosibirsk, Russia; Institute of Cytology and Genetics of the Siberian Branch of the Russian Academy of Sciences, Novosibirsk, Russia Kurchatov Genomic Center of ICG SB RAS, Novosibirsk, Russia; Institute of Cytology and Genetics of the Siberian Branch of the Russian Academy of Sciences, Novosibirsk, Russia Institute of Computational Mathematics and Mathematical Geophysics of the Siberian Branch of the Russian Academy of Sciences, Novosibirsk, Russia; Institute of Cytology and Genetics of the Siberian Branch of the Russian Academy of Sciences, Novosibirsk, Russia Novosibirsk State University, Novosibirsk, Russia Kurchatov Genomic Center of ICG SB RAS, Novosibirsk, Russia; Institute of Cytology and Genetics of the Siberian Branch of the Russian Academy of Sciences, Novosibirsk, Russia; Institute of Cytology and Genetics of the Siberian Branch of the Russian Academy of Sciences, Novosibirsk, Russia Novosibirsk State University, Novosibirsk, Russia; Institute of Cytology and Genetics of the Siberian Branch of the Russian Academy of Sciences, Novosibirsk, Russia Kurchatov Genomic Center of ICG SB RAS, Novosibirsk, Russia

**Keywords:** human, TBP, SNP, promoter, hub gene, SCN9A, expression change, pain generation, pain perception, pain response, anesthesia, человек, ТВР, SNP, промотор, ген-интегратор, SCN9A, изменение экспрессии, генерация боли, чувство боли, ответ на боль, анестезия

## Abstract

In this work, we for the first time performed a comprehensive bioinformatics analysis of 568 human genes that, according to the NCBI Gene database as on September 15, 2024, were associated with pain generation, perception and anesthesia. The SCN9A gene encoding the sodium voltage-gated channel α subunit 9 and expressed in sensory neurons for transferring signals to the central nervous system about tissue damage was the only one involved in all the processes of interest at once as a hub gene. First, with our tool called OrthoWeb, we estimated the phylostratigraphic age indices (PAIs) for each of the genes, that is, identified the taxon of the most recent common ancestor of the organisms for which that gene has been sequenced. The mean PAI for all genes under study, including SCN9A as a hub gene for pain generation, perception, response and anesthesia, was ‘4’. On the evolutionary scale by the Kyoto Encyclopedia of Genes and Genomes (KEGG), the ancestor is the phylum Chordata, some of the most ancient of which evolved the central and the peripheral nervous system. Next, with our tool called ANDSystem, we found that phosphorylation of ion channels is a centerpiece in pain generation, perception, response and anesthesia, on which the efficiency of signal transduction from the peripheral to the central system depends. This conclusion was consistent with literature data on a key role an efficient signal transduction from the peripheral to the central system from the peripheral to the central system for adjusting the human circadian rhythm through detection of a change from the dark of night to the light of day and for identification of the direction of the source of sound by auditory brainstem nuclei, for generating the response to cold stress and for physical coordination. 21 candidate SNP marker of significant SCN9A over- and underexpression. Finally, the ratio of SCN9A upregulating to downregulating SNPs was compared to that for all known human genes estimated by the 1000 Genomes Project Consortium. It was found that SCN9A as a hub gene for pain generation, perception, pain response and anesthesia is acted on by natural selection against its downregulation, to keep the nervous system highly informed on the status of the organism and the environment.

## Abstract (ru)

В настоящей работе впервые проведен комплексный биоинформатический анализ генов человека, связанных с генерацией, чувством и ответом на боль наряду с обезболиванием, которые были представлены 568 генами человека согласно базе данных NCBI Gene (дата обращения 15.09.2024). Ген SCN9A человека (sodium voltage-gated channel α subunit 9) передачи сигналов о повреждении тканей от сенсорных нейронов в центральную нервную систему был единственным среди исследуемых 568 генов, который вовлечен во все анализируемые процессы как ген-интегратор для них. Сначала с использованием созданного нами ранее инструмента OrthoWeb для каждого гена оценили таксон ближайшего общего предка всех организмов, у которого расшифрована ДНК этого гена (т. е. индекс филостратиграфического возраста, PAI). Среднеарифметическая оценка PAI для всех анализируемых генов, а также его значение для гена SCN9A, интегратора генерации, чувства и ответа на боль наряду с анестезией, оказались равными 4. На эволюционной шкале Киотской энциклопедии генов и геномов (KEGG) это соответствует таксону Chordata, у одних из самых древних представителей которого произошла специализация центральной и периферической нервной системы. Далее с помощью созданной нами системы ANDSystem мы выявили фосфорилирование ионных каналов как краеугольного камня в генерации, чувстве, ответе на боль и обезболивание, которое определяет эффективность передачи сигналов из периферической в центральную нервную систему. Этот вывод согласуется с литературными данными о ключевой роли эффективной передачи сигналов периферической нервной системы в центральную при коррекции циркадного ритма человека через фактическую детекцию фоторецепторами смены ночной темноты на дневное освещение, а также при определении направления на источник звука слуховыми ядрами мозга, формировании ответа на холодовой стресс и при координации движений у человека. Затем с использованием ранее созданной нами базы данных Human_SNP_TATAdb был предложен 21 кандидатный SNP-маркер значимого увеличения и уменьшения экспрессии гена SCN9A человека. Наконец, отношение встречаемости этих SNP-маркеров сравнили с полногеномным отношением, которое было оценено консорциумом «1000 геномов». В результате обнаружено, что SCN9A как ген-интегратор генерации, чувства, ответа на боль наряду с анестезией подвержен естественному отбору против снижения его экспрессии для поддержания высокого уровня контроля состояния организма и параметров внешней среды.

## Introduction

In 2020, the Council of the International Association for the Study of Pain (IASP) unanimously accepted the definition of pain as “An unpleasant sensory and emotional experience associated with, or resembling that associated with, actual or potential tissue damage” (Raja et al., 2020). Six accompanying notes were accepted to ensure the proper use of the term pain depending on the context (Raja et al., 2020). It was recommended that pain be conceived as an individual’s unpleasant emotional experience enhanced by biological, psychological and social factors. In addition, pain is not the same as the pulsing activity of the peripheral and the central nervous system’s sensitive nervous fibers excited by diverse stimuli and called “nociception”, “nociperception” or – in a narrower sense – physiological pain. The individuals develop the concept of pain as part of their personal experience. The IASP Council also recommended that the patients’ opinion about the pain they sense be considered. Although pain serves an adaptive role, it may have an adverse effect on social and psychological well-being as well as on the function of the human organism. Finally, the verbal description of pain is one of the many ways the individual can express this feeling and if he fails, chances are he may be is experiencing it nonetheless.

Considering the above, we focused on physiological pain, to be termed just “pain” throughout for brevity and because the term pain is used in this narrow sense by such renowned sources of scientific data as NCBI Gene (Brown et al., 2015) and Gene Ontology (Gene Ontology Consortium, 2015), on which we rely in this work.

Here we are for the first time conducting a comprehensive bioinformatics study of pain and anesthesia as a practical service in applied medicine, when patient treatment requires that both self-consciousness and awareness of the environment be reduced or eliminated by use of anesthetic drugs essential for organismic homeostasis, according to recommendations from the Association of Anaesthetists’ (Klein et al., 2021; Lucas et al., 2021). The need to explore further is so high that 49,305 and 3,782 original scientific papers related to pain and anesthesia, respectively, were collected in PubMed (Lu, 2011) as on September 15, 2024. With this in mind, we used our freely available web services OrthoWeb (Mustafin et al., 2020) and ANDSystem (Ivanisenko et al., 2015), and the Human_SNP_TATAdb database (Filonov et al., 2023) and analyzed 568 human genes associated with pain generation, perception, response and anesthesia, according to NCBI Gene (Brown et al., 2015) as on September 15, 2024. We verified our results against data from the independent web services PANTHER (Mi et al., 2021), DAVID (Sherman et al., 2022), STRING (Szklarczyk et al., 2023), Metascape (Zhou et al., 2019) and GeneMania (Warde-Farley et al., 2010), the ClinVar database (Landrum et al., 2014) and similar whole-genome results coming from the 1000 Genomes Project Consortium (1000 Genomes Project Consortium et al., 2012), with Haldane’s dilemma (Haldane, 1957) and the neutral theory of molecular evolution (Kimura, 1968) factored in.

## Materials and methods

The human genes. A total of 568 human genes (n = 568) were studied. The list of the genes was generated by querying “Homo sapiens” AND “[gene key word]” in NCBI Gene (Brown et al., 2015) accessed on September 15, 2024. The activated filters were Protein-coding genes, Genomic, Annotated genes, Ensembl and Current, to return the most completely annotated protein-coding human genes

The Phylostratigraphic Age Index (PAI) of the human genes. With OrthoWeb (Mustafin et al., 2020), we identified for each of the 568 genes all the biological species that had freely available orthologs to this gene and thus identified the most recent common ancestor of these species (Samet, 1985; Sun et al., 2008; Morozova et al., 2020), whose age served as the phylostratigraphic age indices (PAI) of the gene according to KEGG, the Kyoto Encyclopedia of Genes and Genomes (Kanehisa, Goto, 2000).

The associative network for pain generation, perception, response and anesthesia was reconstructed using ANDSystem (Ivanisenko et al., 2015). The results obtained were verified against the independent web services PANTHER (Mi et al., 2021), DAVID (Sherman et al., 2022), STRING (Szklarczyk et al., 2023), Metascape (Zhou et al., 2019) and GeneMania (Warde-Farley et al., 2010). The amount of consistency between the results coming from these web service and ANDSystem (Ivanisenko et al., 2015) was inferred by searching for the corresponding publications in PubMed (Lu, 2011).

Supervised annotation of the effects of changes in human gene expression levels on pain generation, perception, response and anesthesia. The effects of changes in SCN9A expression levels on pain generation, perception, response and anesthesia were explored by searching for the corresponding publications in PubMed (Lu, 2011).

The effects of single-nucleotide polymorphism (SNP) variants in the human gene promoters on the expression levels of these genes. The estimates of the statistical significance of the decrease or increase in the expression levels of the human genes for the minor vs. reference alleles of the SNP in the promoters of these genes were taken from the Human_SNP_TATAdb knowledge base (Filonov et al., 2023).

Verification of the estimations of the effects of SNPs in the human gene promoters on the expression levels of these genes. Selective verification of the in silico estimates of the effects of SNPs in the human gene promoters on the expression levels of these genes was performed using ClinVar (Landrum et al., 2014), PubMed (Lu, 2011) and literature data by the 1000 Genomes Project Consortium (Lowy-Gallego et al., 2019) for assessing the prevalence of such SNPs in the entire reference human genome, with Haldane’s dilemma (Haldane, 1957) and the neutral theory of molecular evolution (Kimura, 1968) factored in.

Statistical analysis. The statistical criteria for the Kolmogorov– Smirnov test and the binomial distribution were tested using STATISTICA (Statsoft ^ ™^ USA).

## Results

SCN9A as a hub gene for pain generation, perception, response and anesthesia

**Fig. 1.**
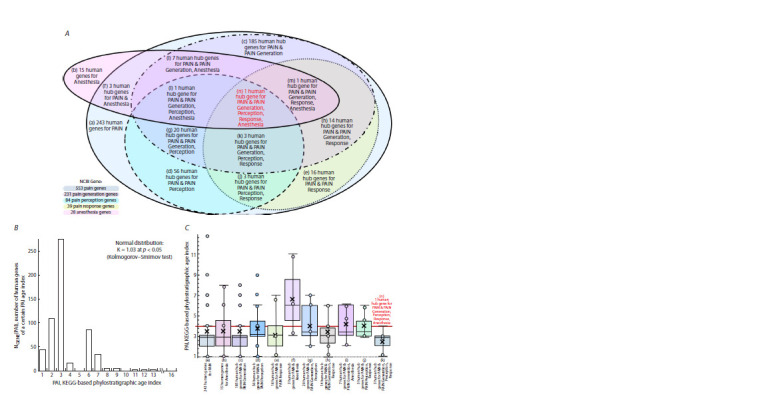
The human genes returned by querying “Homo sapiens” AND “[gene key word]” in NCBI Gene (Brown et al., 2015) with Protein-coding, Genomic, Annotated genes, Ensembl and Current as activated filters.

We have herein worked on 568 human genes selected with NCBI Gene (Brown et al., 2015) (see Materials and methods). Of them, 553 were associated with pain; 231, with pain generation; 84, with pain perception; 39, with pain response; and 28, with anesthesia (Fig. 1А). The gene that is in red color font on the Venn diagram showing all possible overlaps between the gene groups (Fig. 1А) is SCN9A, the only gene shared by these groups. SCN9A encodes the sodium voltage-gated channel α subunit 9 and is expressed in sensory neurons for transferring signals to the central nervous system about tissue damage. Thus it was decided to consider SCN9A to be a hub gene for pain generation, perception, response and anesthesia.

The differences in PAI between the pain-generationspecific, perception-specific, response-specific and anesthesia-specific groups of genes do not reach statistical significance

We estimated the phylostratigraphic age index (PAI) for each of the 568 human genes. The histogram with the number of the genes being worked with within each of the 16 time intervals on the PAI scale according to the Kyoto Encyclopedia of Genes and Genomes (KEGG) (Kanehisa, Goto, 2000) is shown in the Figure 1B. The evolutionary estimates of the PAIs of the human genes associated with pain generation, perception, response and anesthesia, statistically significantly meet a normal distribution (Kolmogorov–Smirnov test: K = 1.03, p < 0.05). In line with the Central Limit Theorem (Kwak, Kim, 2017), which may imply that the PAI estimates reflect an integration of a great diversity of critical pain criteria. Considering this, we focused on SCN9A as a human hub gene for pain generation, perception, response and anesthesia.

The hypothetical link between the PAIs of the human genes associated with either pain generation or perception or response or anesthesia was verified using a box-and-whisker diagram for the overlaps between these groups of genes (Fig. 1C). The difference in PAI between the overlapped portions of the gene groups does not reach statistical significance, nor does it the difference between them and SCN9A as a hub gene for pain criteria in humans (Fig. 1А). That fact reinforced our confidence that SCN9A is worthy of our commitment.

The associative network for pain generation, perception, response and anesthesia

**Fig. 2.**
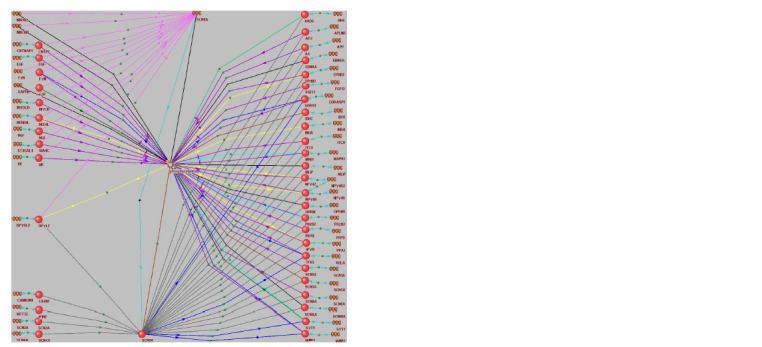
The associative network of SCN9A, its encoded protein and their closest partners in the human organism. The network was constructed with ANDSystem (Ivanisenko et al., 2015) by automated analysis of freely available texts and database entries returned by querying “[list of genes] [immediate associations only] Genes Proteins Pathway ” for [list of genes] = “SCN9A”. Legend: – gene; ● – protein; – phosphorylation as the most statistically significant biological process involving all the genes and proteins found (PADJ < 10–13, Fisher’s Z with the Bonferroni correction for multiple comparisons). Arrows: sharp-headed – activation; bluntheaded – inhibition; head-free – involvement; yellow – activity; dark-blue – transport; black – contact; purple – function; red – regulation; turquoise – expression.

The associative network for SCN9A (Fig. 2) was constructed with ANDSystem (Ivanisenko et al., 2015). In the upper central part is the human gene SCN9A; in the lower central part, its encoded protein; in the middle central part, phosphorylation as a molecular-genetic process that is most mentioned in relation to this gene, as ANDSystem (Ivanisenko et al., 2015) suggests.

In the left-hand central part of the Figure 2 is DPYSL2, the only human gene associated with SCN9A itself, its encoded protein and phosphorylation. Additionally, in the left-hand bottom corner are four genes and their encoded proteins that interact with SCN9A, and in the left-hand upper corner are 11 human genes and their encoded proteins that interact with SCN9A and are involved in phosphorylation. The other 25 genes and their proteins interact with SCN9A and are involved in phosphorylation, too (Fig. 2, right). In total, Figure 2 shows 42 human genes, of which 14 were among the 568 genes associated with pain generation, perception, response and anesthesia (Fig. 1).

The overlap between the lists of 42 and 568 genes is statistically significant in terms of the reference human genome, which contains 19,424 annotated protein-coding genes, as suggested by NCBI Gene (Brown et al., 2015) as on August 20, 2024, with Ensembl, Current, Protein-coding genes, Genomic and Annotated genes as the activated filters: the binomial distribution at p < 10–6.

This implies that ANDSystem (Ivanisenko et al., 2015) fed with SCN9A alone as a hub gene for pain generation, perception, response and anesthesia (Fig. 1) statistically significantly reconstructed the list of human genes (Fig. 2) that are associated with these processes in NCBI Gene (Brown et al., 2015).

Verification of the ANDSystem result against those on Gene Ontology term enrichment for the groups of genes by independent web services

A comparison between the result by ANDSystem (Ivanisenko et al., 2015) suggesting that phosphorylation is the most statistically significant biological process for pain generation, perception, response and anesthesia (Fig. 2) and the results by independent web services on Gene Ontology term enrichment for the groups of genes (Gene Ontology Consortium, 2015) is given in Table 1.

**Table 1.**
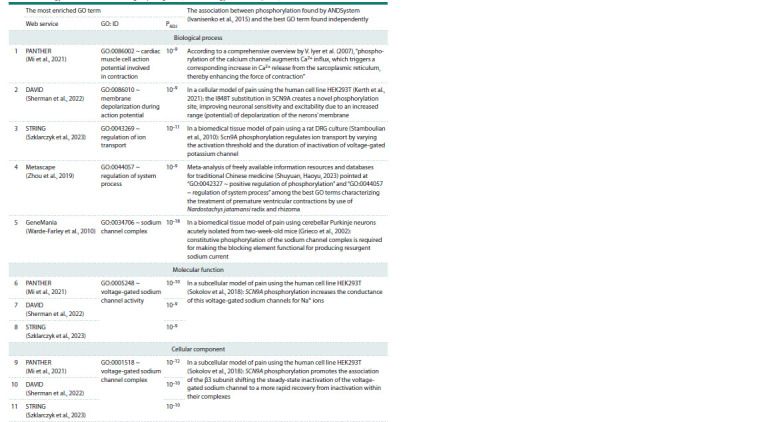
A comparison between the result by ANDSystem (Ivanisenko et al., 2015) and the results by other web services on Gene Ontology term enrichment for the groups of genes (Gene Ontology Consortium, 2015) Note. PADJ is the statistical significance of GO term enrichment for the groups of genes, with a correction for multiple comparisons used in the web service as indicated.

For example, as the upper row of that table suggests, for 42 human genes in the Figure 2, the web service PANTHER (Mi et al., 2021) revealed “GO:0086002 ~ cardiac muscle cell action potential involved in contraction” as the most statistically significant biological process involving these 42 genes (PADJ <10–9, statistical significance with a correction for multiple comparisons). The rightmost cell of this row contains a citation from an overview by V. Iyer et al. (2007): “Phosphorylation of the calcium channel augments Ca2+ influx, which triggers a corresponding increase in Ca2+ release from the sarcoplasmic reticulum, thereby enhancing the force of contraction”. In its sense, the results from ANDSystem (Ivanisenko et al., 2015) and PANTHER (Mi et al., 2021) for the 42 genes in Figure 2 are consistent.

In total, Table 1 shows 11 similar consistencies between the results coming from ANDSystem and five independent web services (PANTHER, DAVID, STRING, Metascape and GeneMania) about GO term enrichment for the groups of gene (Gene Ontology Consortium, 2015).

The effects of changes in the expression levels of SCN9A as a hub gene on pain generation, perception, response and anesthesia

At this stage of our work, we sent text-based queries to PubMed (Lu, 2011) and thus performed a supervised annotation of SCN9A down- and upregulation by comparing them with literature data on the clinical manifestations of the changes in pain generation, perception, response and anesthesia (Table 2).

**Table 2.**
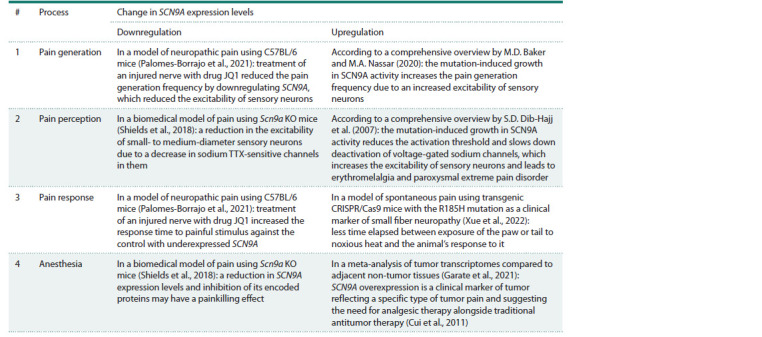
Clinical implications of SCN9A downregulation and upregulation for pain generation, perception, response and anesthesia according to PubMed (Lu, 2011)

**Fig. 3.**
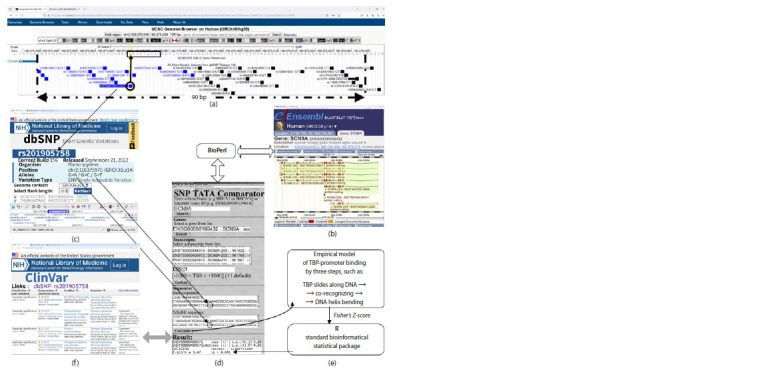
An example: analysis of the candidate SNP marker rs201905758:T in the 90-bp proximal region (a two-headed dash-and-dot arrow in pane (а) before the start site of transcript SCN9A-203 from SCN9A, according to Ensembl (Zerbino et al., 2015), using SNP_TATA_Comparator (Ponomarenko et al., 2015). Legend: (а) – visualization of the promoter being analyzed with the web service UCSC Genome Browser (Raney et al., 2024); (b) – the Ensembl database (Zerbino et al., 2015); (c) – description of SNP rs201905758 in the dbSNP database (Day, 2010); (d) and (e) – the use of SNP_TATA_Comparator and the principle of its operation, respectively (Ponomarenko et al., 2015); (f ) – description of rs201905758:G→t, a clinically proven SNP marker for pain sensing pathology, according to ClinVar (Landrum et al., 2014).

In Human_SNP_TATAdb (Filonov et al., 2023), we found 21 candidate SNP marker of a significant change in TBP affinity for the promoters of this gene and, consequently, a change in the expression levels of this gene (Table 3). Four of the 21 SNP marker of the significant change in SCN9A expression levels have known clinical implications (Table 3), as ClinVar (Landrum et al., 2014) suggests. It was demonstrated, with one of the four clinical SNP markers of pain, rs201905758:T as an example, (Fig. 3), how this SNP marker was detected by the web service SNP_TATA_Comparator (Ponomarenko et al., 2015) run in automated mode using the BioPerl library (Stajich et al., 2002) for access to Ensembl (Zerbino et al., 2015) and dbSNP (Day, 2010), the official repository of the reference human genome and the reference human variome, respectively. According to ClinVar (Landrum et al., 2014), four of the 21 SNPs were clinically proven markers of paroxysmal extreme pain disorder (PEPD), small fiber neuropathy (SFN), primary erythromelalgia (PE) and channelopathy-associated congenital insensitivity to pain (CIP) (Table 3).

**Table 3.**
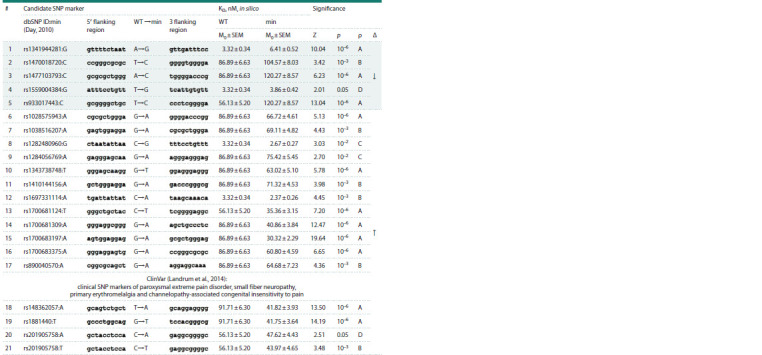
Candidate SNP markers in the 90-bp proximal regions of the promoters before the transcription start sites of SCN9A, a human gene for pain integration, generation, perception, response and anesthesia, according to our in silico analysis as shown in the Figure 3 and documented in the Human_SNP_TATAdb database (Filonov et al., 2023) Note. WT and min are the ancestral (norm) and the minor (pathology) alleles of the SNP, respectively; KD is the equilibrium dissociation constant of the TBP–promoter complex expressed in nanomoles per liter (nM); M0 and SEN are the context-dependent in silico estimate and its standard error, respectively; Z, p and ρ are the Fisher Z value and the level of its statistical significance as well as the heuristic prioritization of the in silico estimates from the best (А) to the worst (D) in alphabetic order; Δ – increase (↑) or decrease (↓) in SCN9A expression levels.

As can be seen from the rightmost column “Δ” of Table 3, any of these four clinically proven markers of the SCN9A gene increases its expression levels as a hub gene for pain generation, perception, response and anesthesia. This encouraged us to perform a supervised PubMed-based annotation of the effects of SCN9A overexpression on pain generation, perception, response and anesthesia (Table 4). According to the many clinical overviews that have been written, for example (Dabby, 2012; Bennett, Woods, 2014; Shields et al., 2018; Taub, Woolf, 2024), SCN9A excess in PEPD, SFN and PE increases pain generation, perception and response, while low-molecular-weight inhibitors of SCN9A are anesthetics.

**Table 4.**
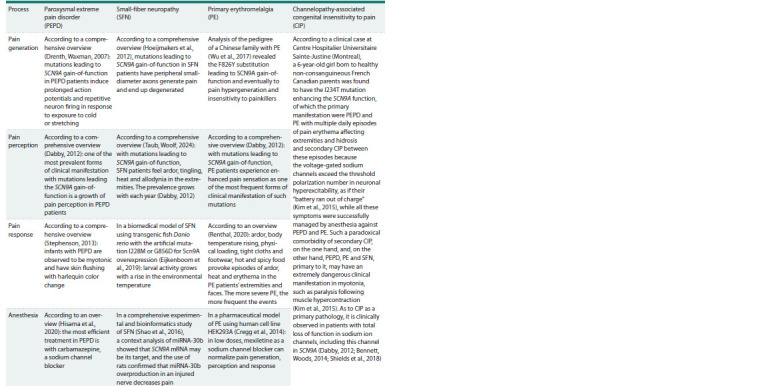
The effect of SCN9A overexpression on pain generation, perception and anesthesia in paroxysmal extreme pain disorder, primary erythromelalgia, small-fiber neuropathy and channelopathy-associated congenital insensitivity to pain, according to PubMed (Lu, 2011)

As far as CIP is concerned, according to clinical observations (Kim et al., 2015), secondary insensitivity to pain alternates with episodes of hypersensitivity to pain in PEPD, SFN and PE due to excessive SCN9A, this hypersensitivity being primary to insensitivity. It looks as if, because there were too many voltage-gated sodium channels in SCN9A, neural hyperexcitability depleted their battery now it needs to be recharged – to recover the previous levels of pain generation, perception and response. In this sense, all the in silico estimates of SCN9A overexpression with all clinically proven SNP markers of pain in PEPD, SFN, PE and CIP are consistent with the manifestation of excessive SCN9A in patients with these pathologies.

Comparison of the prevalence of the candidate SNP markers of changes in SCN9A expression levels against the whole-genome frequency of such SNPs

In conclusion, we compared the prevalence of the candidate SNP markers of changes in SCN9A expression levels (Table 3) with the frequency of such SNPs across the human genome according to 1000 Genomes Project (Table 5). Individual human genomes possess an average of 1,000 SNPs each, of which 200 and 800 correspond, respectively, to an increase and a decrease in ТВР-promoter affinity and eventually и to an increase and a decrease in the expression levels of human genes with these SNPs (Kasowski et al., 2010; 1000 Genomes Project Consortium et al., 2012).

**Table 5.**
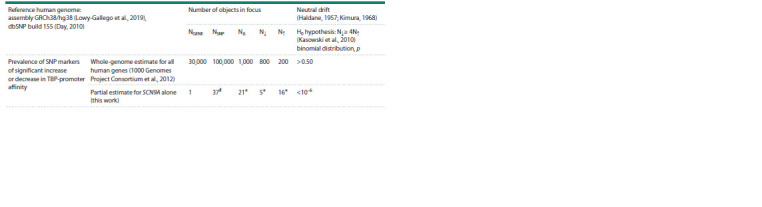
A comparison between the prevalence of the identified candidate SNP markers of increased and decreased affinity of ТВР to the SCN9A promoters (Fig. 3, Table 3) against whole-genome estimates according to the 1000 Genomes Project Note. NGENE – number of genes being worked with; NSNP – number of SNPs being worked with; NΔ – number of SNPs with ability to increase (N↓) and to decrease (N↑) ТВР affinity to human gene promoters. # – see Figure 3, А; * – see Table 2.

In terms of Haldane’s dilemma (Haldane, 1957) and the neutral theory of molecular evolution (Kimura, 1968), this prevalence of deleterious over beneficial regulatory SNPs signifies a neutral drift event, whish is statistically significantly different from the prevalence of 21 candidate SNP marker of changes in SCN9A expression levels (p < 10–6, binomial distribution) (Table 5). This result implies that SCN9A is under natural selection against its downregulation, to keep the nervous system highly informed on the status of the organism and the environment.

## Discussion

In this work, we for the first time performed a comprehensive bioinformatics analysis of 568 human genes that, according to the NCBI Gene database as on September 15, 2024, were associated with pain generation, perception and anesthesia. Our effort was strongly enabled by our freely available developments OrthoWeb (Mustafin et al., 2020), ANDSystem (Ivanisenko et al., 2015) and Human_SNP_TATAdb (Filonov et al., 2023). As a result, we identified SCN9A as being a hub gene for these biological processes (Fig. 1). Its Phylostratigraphic Age Index PAI = 4, according to the KEGG scale (Kanehisa, Goto, 2000), was not statistically different from the PAIs of the human genes associated with any of the combinations of the pain-related conditions in question and corresponded to the phylum Chordata, some of the most ancient of which evolved the central and the peripheral nervous system (Holland L.Z., Holland N.D., 1999).

Phosphorylation was found to be a key molecular genetic process in pain generation, response and anesthesia (Fig. 2). This result is consistent, first of all, with experimental data for a biomedical model of pain using the human cell line HEK293T (Kerth et al., 2021). C.M. Kerth and the co-workers found that the I → T substitution at position 848 of human protein SCN9A creates a novel phosphorylation site of this protein, which is accompanied by an increase in neuronal sensitivity and excitability due to an increased range (potential) of depolarization of the neurons’ membrane

Additionally, the conclusion made about the importance of ion channel phosphorylation for pain generation, response and anesthesia is consistent to (Table 1) literature data about the importance of calcium channel phosphorylation in the myocardial cells (Iyer et al., 2007) and the importance of sodium channel phosphorylation in the cerebellar Purkinje neurons for physical coordination (Grieco et al., 2002).

Another example was found in PubMed (Lu, 2011): a cellular model of circadian rhythm using chick photoreceptors; under this model, increased phosphorylation of the ion channels in retinal cones in response to increased illumination the day offers after the dark of the night was the main event of the circadian rhythm in this model animal (Chae et al., 2007). The study of ophthalmic pathologies in rats revealed that phosphorylation of the ion channels in the optic nerve regulates visual system pathways (Ogata et al., 2022). Additionally, phosphorylation of the potassium channel in auditory neurons is basic to the ability to identify the direction of the source of sound due to microsecond delays in registering signals from it by auditory brainstem nuclei (Song et al., 2005). The phosphorylation levels of the SNAP-25 channel in the amygdala, cortex and hippocampus increased with the growth in the intensity of cold stress in mouse studies (Yamamori et al., 2014). Together, this provides a solid piece of evidence about a key role that ion channel phosphorylation has in the specialization into the central and the peripheral nervous system in general and during pain generation, perception, response and anesthesia.

At the final step, we used the Human_SNP_TATAdb database (Filonov et al., 2023) and proposed 21 candidate SNP marker of a significant change in the expression levels of SCN9A which encodes the sodium voltage-gated channel α subunit 9 and is expressed in sensory neurons for transferring signals to the central nervous system about tissue damage (Table 3). In ClinVar (Landrum et al., 2014), we found the descriptions of clinical in vivo manifestations for four of the 21 predicted SNP markers that were consistent with our in silico estimates (Table 4). A comparison between the prevalence of the SNPs identified in the SCN9A promoters and the whole-genome estimates according to the 1000 Genomes Project Consortium in 2012 leads to the conclusion that natural selection acts against SCN9A downregulation (Table 5), which indicates an adaptive role of pain and its perception as well as response to pain and anesthesia (Raja et al., 2020).

Overall, the results obtained are consistent with the independent authors’, and in some cases refine and summarize them.

## Conclusion

We have for the first time performed a comprehensive bioinformatics analysis of 568 human genes, which according to the NCBI Gene database (Brown et al., 2015) were associated with pain and anesthesia. From among them, we singled out SCN9A, the gene encoding the sodium voltage-gated channel α subunit 9 and expressed in sensory neurons for transferring signals to the central nervous system about tissue damage was the only one involved in all the processes of interest at once as a hub gene. With OrthoWeb (Mustafin et al., 2020), we estimated the phylostratigraphic age index (PAI) for SCN9A. It was “4”, which corresponds to the phylum Chordata, some of the most ancient of which evolved the central and the peripheral nervous system (Holland L.Z., Holland N.D., 1999). The associative network of SCN9A was reconstructed using ANDSystem (Ivanisenko et al., 2015), where ion channel phosphorylation in SCN9A is a factor on which the efficiency of signal transduction from the peripheral to the central nervous system depends and which is a centerpiece in pain generation, perception, response and anesthesia. Finally, the search of the Human_SNP_TATAdb database (Filonov et al., 2023) revealed 21 candidate SNP marker of a significant change in SCN9A expression levels. The ratio of SCN9A upregulating to downregulating SNPs was compared to that for all known human genes (1000 Genomes Project Consortium et al., 2012). As a result, we for the first time obtained in silico whole-genome evidence that pain generation, perception, response and anesthesia (Raja et al., 2020) have an adaptive role, and their efficiency is controlled by natural selection.

## Conflict of interest

The authors declare no conflict of interest.
